# KinectGaitNet: Kinect-Based Gait Recognition Using Deep Convolutional Neural Network

**DOI:** 10.3390/s22072631

**Published:** 2022-03-29

**Authors:** A. S. M. Hossain Bari, Marina L. Gavrilova

**Affiliations:** Department of Computer Science, University of Calgary, Calgary, AB T2N 1N4, Canada; mgavrilo@ucalgary.ca

**Keywords:** deep convolutional neural network, hierarchical feature extraction, resampling, kinect-based gait recognition, behavioral biometric

## Abstract

Over the past decade, gait recognition had gained a lot of attention in various research and industrial domains. These include remote surveillance, border control, medical rehabilitation, emotion detection from posture, fall detection, and sports training. The main advantages of identifying a person by their gait include unobtrusiveness, acceptance, and low costs. This paper proposes a convolutional neural network KinectGaitNet for Kinect-based gait recognition. The 3D coordinates of each of the body joints over the gait cycle are transformed to create a unique input representation. The proposed KinectGaitNet is trained directly using the 3D input representation without the necessity of the handcrafted features. The KinectGaitNet design allows avoiding gait cycle resampling, and the residual learning method ensures high accuracy without the degradation problem. The proposed deep learning architecture surpasses the recognition performance of all state-of-the-art methods for Kinect-based gait recognition by achieving 96.91% accuracy on UPCV and 99.33% accuracy on the KGB dataset. The method is the first, to the best of our knowledge, deep learning-based architecture that is based on a unique 3D input representation of joint coordinates. It achieves performance higher than previous traditional and deep learning methods, with fewer parameters and shorter inference time.

## 1. Introduction

Human gait is the repeated pattern of dynamic motions exhibited by the different body joints [1]. Recurrent stances of heel strike, standing, and heel off are exhibited during walking [2]. Unique characteristics extracted from the recurrent locomotion of the body joints are exploited in the biometrics for the identification of a person [1]. The general acceptability of obtaining gait from a distance, low cost, and variety of data acquisition sensors, and in general, a high accuracy of identification of a person from a distance make gait recognition one of the most popular behavioral biometrics [3]. Gait recognition has numerous applications, such as person identification [4,5], human activity recognition [6], gender recognition [7], emotion recognition from human posture [8], search and rescue operations [9,10], access control [11], medical diagnosis, treatment, and rehabilitation [12,13].

Supervised machine learning models trained with distinctive features extracted from the biometric trait pave the way to automate the simulation of the biometric identification [14,15]. Gait-based person identification with the help of traditional machine learning models have been studied considerably over the past decade [16]. The accelerated pace of the development of the powerful deep learning methods has opened up unprecedented opportunities to leverage them in many domains. Domains of computer vision, computational intelligence, cognitive architectures, human–computer interaction, trustworthy decision making, defense, robotics, and biometrics benefit from the development of powerful deep learning architectures that are lightweight and versatile, and they provide high performance without overfitting. Performance of the image classification [17], face recognition [18], facial expression recognition [19], person verification [20], and others [21,22] are enhanced, exploiting the power of deep learning. However, such approaches have been in their infancy in the biometric domain, and they have been concerned with Kinect-based person identification based on human gait [16].

One of the first successful works that introduced deep learning neural network architecture for Kinect-based gait recognition appeared in [23]. Aside from the deep learning approach, there have been many successful approaches devised over the past decade that facilitate the successful recognition of humans based on Kinect-based gait biometrics [24,25,26]. These approaches had a number of deficiencies. Handcrafted classifying features were proposed for Kinect-based gait recognition in [5,23,25,26]. The extraction of handcrafted features requires specialization in the target domain and the selection of uncorrelated distinctive features is difficult to perform. In addition, traditional machine learning methods for gait recognition relied on the computationally expensive pre-processing steps and expensive feature selection methods. A deep convolutional neural network provides new opportunities to overcome the above challenges and thus to improve recognition performance. However, when a deep convolutional network is considered for feature extraction and recognition purposes simultaneously, degradation problems may arise because the error rates in training are increased after the convergence [27,28].

Microsoft Kinect produces a color-based depth video frame with the human skeleton from the 2D color image. The proposed architecture KinectGaitNet addresses the aforementioned challenges while overcoming degradation problems typical for deep convolutional neural networks. The main contributions of the proposed method can be outlined as follows. First, a unique 3D input representation of joint coordinates during the gait cycle is proposed. Thus, without extracting handcrafted features, the proposed input representation serves as the input of the CNN architecture for hierarchical feature extraction. Second, a new convolutional neural network architecture called KinectGaitNet based on residual learning is designed. Two types of residual learning blocks are introduced in such a way that the degradation problem is mitigated and the number of trainable parameters does not increase. Third, the KinectGaitNet architecture is being trained on variable length gait cycles, without the need of resampling to a fixed length. This is accomplished by the introduction of the global average pooling layer before the decision layer. Finally, the Adam optimization method [29] is applied to optimize the weights of the KinectGaitNet for training the model faster and providing robustness to the model, which works with the adaptive learning rate. Two publicly available benchmark datasets, the UPCV gait dataset [7] and Kinect Gait Biometry dataset [24], are used to evaluate the performance of the proposed method.

## 2. Literature Review

The Microsoft Kinect sensor is well suited for indoor and outdoor environments because of the markerless motion analysis, easy accessibility of sensor data, and cost-effectiveness. The Kinect sensor can generate 3D skeleton data at the speed of 30 frames per second [30]. Moreover, the extraction of the body joints tracked by the Kinect sensor shows the accuracy and precision of less than 2 mm [31]. Clark et al. [32] validated the applicability of the Kinect sensor for gait analysis by conducting experiments on the kinectmatic, postural, and spatiotemporal analysis.

The work on model-based gait recognition using the Kinect sensor was started by Preis et al. [4], who introduced eleven handcrafted static and two dynamic features with Rule-based, Decision Tree (DTree), and Naïve Bayes classifiers. In the same year, temporal features of eighteen angles calculated from the selected body joints were extracted to investigate the gait attributes using the K-means clustering method [33]. Later, Joint Relative Distance (JRD) and Joint Relative Angle (JRA) features were proposed in [25], and the rank-level fusion technique was applied to fuse those features. Andersson and Araujo [24] applied Multi-Layer Perceptron (MLP) architecture; however, the performance of the K-Nearest Neighbors (KNN) and Support Vector Machine (SVM) classifiers were better than that of MLP architecture. Yang et al. [26] extracted relative distance features from selective body joints and determined their average and standard deviation over the frames of a gait cycle. Recently, by performing feature extraction from selected body joints, Sun et al. [5] extracted static and dynamic features to train the traditional KNN classifier.

From the aforementioned related works, it is evident that prior research relied on handcrafted features to train the traditional classifiers. However, traditional classifiers can not learn hierarchical feature representation from the samples of the input data. In addition, uncorrelated handcrafted feature extraction demands target-specific knowledge. Handcrafted feature extraction also requires a feature selection step to remove features that cause a negative contribution to the performance due to correlation with other features. A first attempt at integrating deep learning with Kinect-based gait recognition was made in 2019. The researchers utilized three hidden layers to design deep neural network [34]. Their method lacked the solution of managing different length gait sequences and thus required an additional step of the majority voting method after determining the prediction labels of each of the frames of a gait sequence. Furthermore, frame-by-frame prediction causes prediction errors because of the similarity of a particular frame with another person’s gait pattern. Another work extracted joint relative distance and joint relative angle features and determined the average and standard deviation of the handcrafted features over 30 frames [35]. Accumulated features were trained using a convolutional neural network and optimized by the Stochastic Gradient Descent optimizer. After the re-implementation of this method, the recognition performance was below 15% on both the UPCV and Kinect Gait Biometry datasets. There are several reasons for low accuracy. First, since the CNN architecture is trained with the handcrafted features, the uniform kernel can not be used to extract hierarchical features using the CNN architecture. Second, the model suffers from overfitting. Third, the gait cycle is not considered for handcrafted features. As a result, this method is not included in the experimental section.

Recently, Bari and Gavrilova [23] proposed a deep learning neural network architecture trained using better hand-engineered Joint Relative Cosine Dissimilarity (JRCD) and Joint Relative Triangle Area (JRTA) features. In [23], the method depends on the performance of JRCD and JRTA features and requires high model parameters because of the neural network design using a multi-layer perceptron approach. In addition, method [23] resamples the gait cycle to make a fixed size feature vector to train the network. In our paper, the aforementioned shortcomings of emerging research on using deep learning for gait recognition are addressed. Our approach avoids the extraction of static and dynamic handcrafted features from selective body joints, and we also establish that features extracted by the improved convolutional neural network can outperform recent state-of-the-art methods using the variable length of the gait cycle among the individuals. Since handcrafted features are not the input of the proposed method, KinectGaitNet is able to utilize a uniform kernel for the convolution to extract distinctive features.

## 3. Proposed Method

The proposed methodology presents several novel contributions. The transformation approach is introduced to transform the coordinates of the body joints into a 2D matrix based on the gait cycle. Then, 2D matrices are merged to create a 3D matrix using *x*, *y*, and *z* coordinates. A Convolutional Neural Network (CNN) is proposed to extract a low-level to high-level distinctive hierarchical feature map and learn a person’s identification from the samples of 3D matrices. Since the input of the CNN architecture is the 3D matrix generated from the body joints, handcrafted features are avoided, and hierarchical features are extracted directly from the body joints. The proposed CNN architecture is designed in such a way that the CNN architecture can handle the variable length of the gait cycles without resampling of the 3D matrix to a fixed dimension. The residual learning [27] blocks are introduced to design the architecture of the CNN model to mitigate the degradation problem with the reduced model parameters. The Adam optimizer is used to minimize the loss of the objective function.

**Figure 1:**
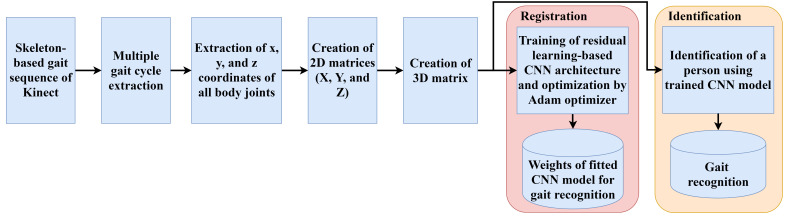
Overall system flowchart of the proposed framework.

There are two phases for the Kinect-based gait recognition. During the registration phase, features are extracted by the proposed convolutional neural network using residual learning from the skeleton-based gait sequences. Extracted features in different layers of the CNN model are trained and optimized during the registration phase. Optimized features are used during the identification phase. During the identification phase, unknown Kinect skeleton-based gait sequences are used. The trained CNN model is applied to the prediction of a person’s identification. The flowchart of the proposed system is shown in Figure 1.

### 3.1. Gait Cycle Detection

**Figure 2:**
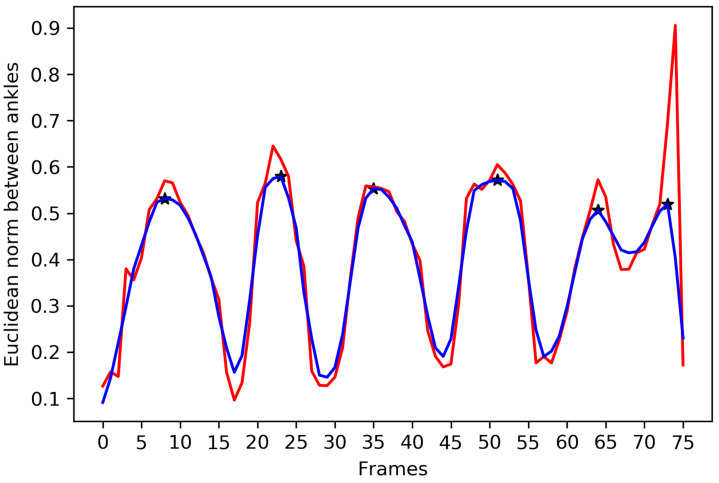
Euclidean norm between ankles is shown in red color. The result of noise reduction filters is shown in blue color. Local maxima is marked using the * symbol.

A cyclic pattern of motion is observed from the body joints of the human body at the time of walking. A gait cycle is detected by tracking the Euclidean distances between two ankles. Since a walking sequence can be affected by the noise, the noise reduction filter is required to detect local maxima to determine a complete gait cycle. First, a moving average filter is applied, and a median filter is further introduced to suppress the noise in the results of the Euclidean norm between two ankles. After the noise reduction, three consecutive local maxima are detected to extract a complete gait cycle. All the local maxima of the noise reduced signal are denoted in Figure 2. The 3D coordinates of each of the body joints of a gait cycle are used to prepare the input of the proposed KinectGaitNet. Each of the gait cycles of a walking sequence exhibits unique gait attributes that need to be extracted and trained using the CNN model.

### 3.2. 3D Matrix Generation from the Body Joint Coordinates

**Figure 3:**
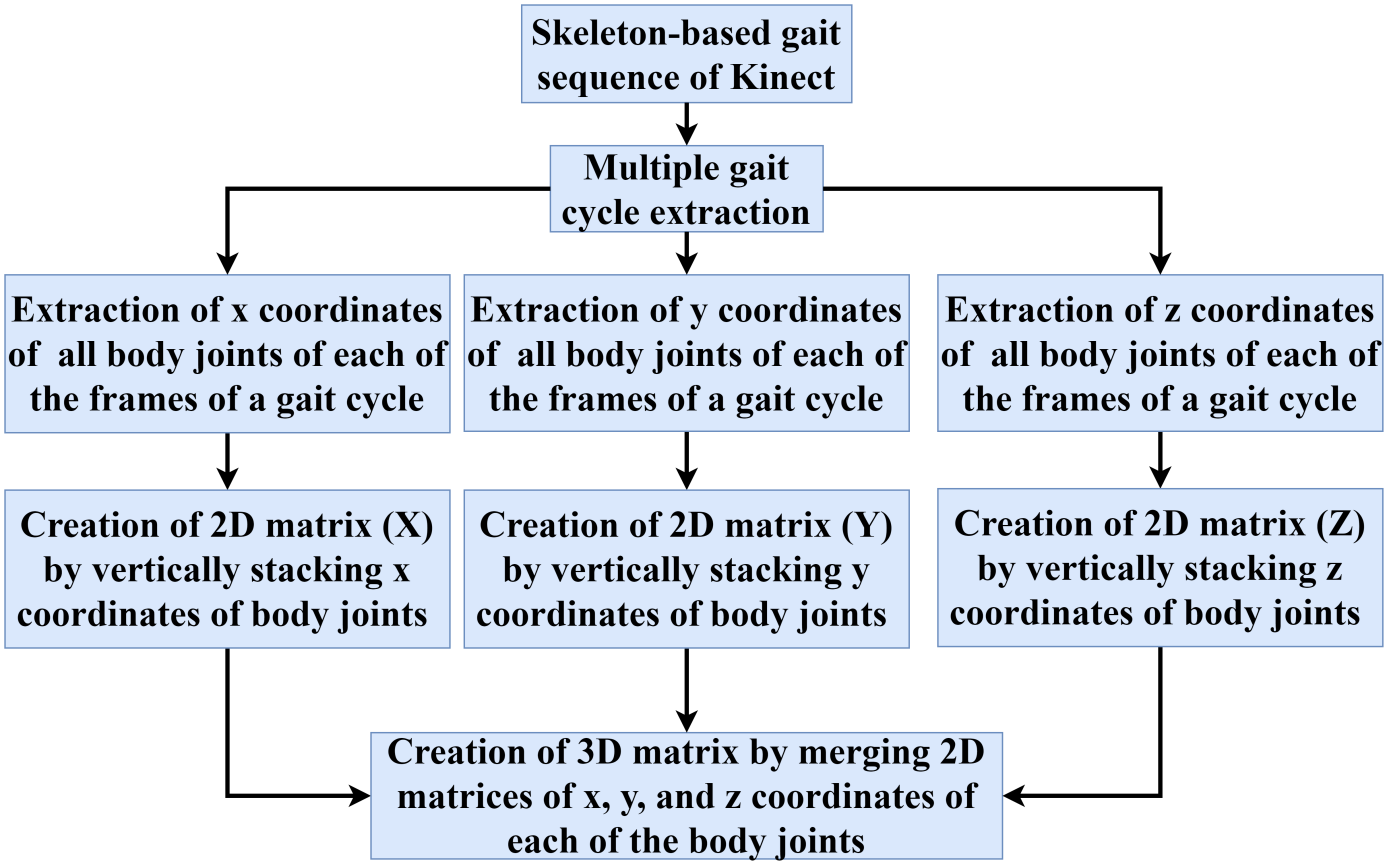
Flowchart of a 3D matrix generation from the body joints over the frames of a gait cycle.

Three-dimensional (3D) coordinates of each of the body joints over a gait cycle are used to generate a unique 3D matrix. Each of the body joints is represented using an (x,y,z) vector in the Kinect skeleton model. The number of body joints and the number of frames in a gait cycle are represented by $N_{b}$ and $N_{f}$, respectively. The *x* coordinates of $N_{b}$ body joints are extracted from each of the frames of a gait cycle. In a similar way, $N_{b}$ number of *y* and *z* coordinates are retrieved. The flowchart of the 3D matrix generation process using the *x*, *y*, and *z* coordinates of each of the body joints over the frames of a gait cycle is shown in Figure 3. Since the number of frames in a gait cycle is not the same for every person, the value of $N_{f}$ is different from person to person.

### 3.3. Proposed Convolutional Neural Network

**Figure 4:**
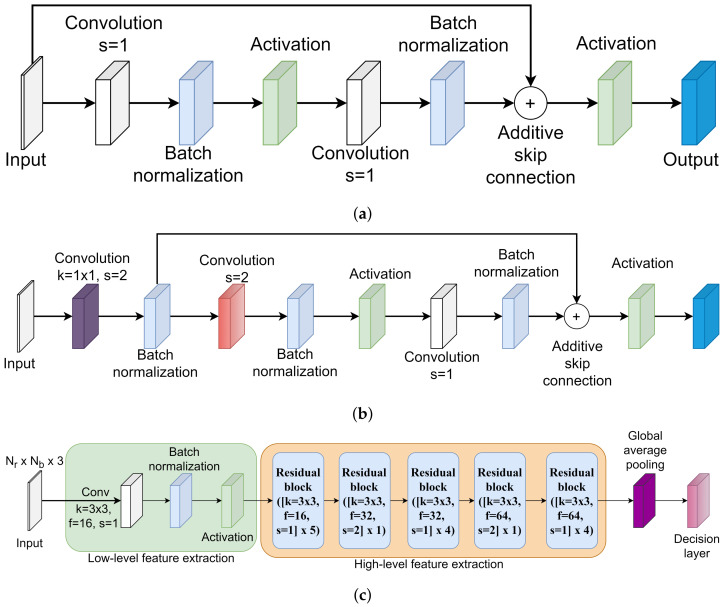
Residual block ([kernel = K×K, filters = *F*, stride = *S*] ×R) means residual blocks are stacked one after another *R* times and K×K kernel of *F* filters are used in the convolutional layer. Based on the value of *S*, one of the residual blocks of Figure 4a,b is selected. (**a**) Architecture of residual block when stride length is set to 1. (**b**) Architecture of residual block when stride length is set to 2. (**c**) Architecture of the KinectGaitNet.

In this paper, a unique residual learning-based convolutional neural network is proposed for the Kinect-based gait recognition. The architecture of the proposed CNN model is shown in Figure 4. The purpose of designing the residual learning-based CNN architecture is to extract hierarchical distinctive features taking the variable dimensions of the 3D matrices as input while avoiding the degradation problem. A 3D matrix comprised of *x*, *y*, and *z* coordinates of each of the body joints over a gait cycle is the input for the proposed CNN architecture. If there are total *N* gait cycles extracted from all persons’ skeleton-based gait sequences, the input shape of the proposed CNN model becomes $N\times N_{f}\times N_{b}\times 3$ where $N_{f}$ is not a fixed value. The identification labels of each of the persons are converted into the one-hot encoded format. If there are total *P* persons’ gait sequences available in a dataset, the shape of the one-hot encoded identification label is N×P. Both the 3D matrix and one-hot encoded identification label are fed into the first layer of the CNN model.

The convolutional layer, batch normalization layer, and activation layer are the first three layers of the proposed CNN model. The spatial and temporal relationships among the body joints and the relationship among *x*, *y*, and *z* coordinates are extracted using the convolutional filters. Extracted features are required to be normalized to make faster convergence of the training with stability. Therefore, the batch normalization layer is subsequently included to transform the extracted features in linear fashion after the convolution layer. The scaled feature map is activated using the Rectified Linear Unit (ReLU) activation. The ReLU activation function is chosen for faster computation, monotonic derivative, reducing the likelihood of vanishing gradient, and faster training. The first three layers are responsible for extracting, scaling, and activating low-level features. Further layers of the KinectGaitNet extract high-level features based on low-level features using residual learning.

There are two types of residual blocks introduced in the proposed architecture in order to extract the hierarchical high-level feature map. The residual block takes the output of the previous layer, size of the kernel, number of filters, and stride length as an input. If the stride length is set to 1 in the residual block, the architecture of the residual block shown in Figure 4a is selected. On the other hand, if the stride length is set to 2 in the residual block, the architecture of the residual block shown in Figure 4b is applied. When the stride length is set to 1, the skip connection is introduced from the input matrix to the results of the batch normalization layer (see Figure 4a). To implement the skip connection, the merging layer of the addition type is used to add the original input matrix to the residual block and the output of the batch normalization layer. The merged results are fed into the ReLU activation layer. When the stride length is set to 2, a convolution operation is applied at first using the provided number of filters with 1×1 kernel. Next, the batch normalization layer is used to normalize the outputs. Consider the result of this batch normalization operation is represented as $Bx_{1}$. The shortcut connection is added from $Bx_{1}$ to the results of the batch normalization layer, according to Figure 4b, using the merging layer of addition type. The merged results are passed to the activation layer. The architectures of Figure 4a,b with skip connection are included in the KinectGaitNet to address the degradation problem, since the high-level feature extraction block is a deeper network.

Traditionally, the result of the final convolutional layer is flattened into the fully connected layer before the decision layer. However, a fully connected layer can not be added after the last residual block because the extracted feature map is in a variable dimension. Since the variable dimension of the 3D matrix is the input of the KinectGaitNet, the dimension of the extracted feature map after the residual block is not consistent for every gait cycle. The feature map needs to be accumulated in such a way that a consistent feature map can be generated and the accumulation process is learnable. To achieve that, we feed the output of the final residual block into a global average pooling layer [36]. The global average pooling layer provides the ability of the KinectGaitNet to support the variable dimension of 3D matrices. It also significantly reduces the number of trainable parameters. Finally, the feature maps are transformed in such a way that the output of the global average pooling operation is closely related to the classification categories.

The softmax activation function is applied at the decision layer to classify persons’ identities in a multi-class gait recognition system. The categorical log loss objective function is optimized using the Adam optimizer to utilize the optimization gain of AdaGrad and RMSProp [37]. Furthermore, the Adam optimizer provides the robustness while optimizing the hyperparameter with an adaptive learning rate.

## 4. Experimental Results

The performance of the proposed KinectGaitNet is evaluated using two publicly available benchmark datasets. The UPCV dataset [7] contains five gait sequences for 30 participants recorded using the Microsoft Kinect sensor at a real-time speed of 30 fps. Among the 30 participants, an equal number of male and female participants contributed to the dataset. The Kinect Gait Biometry (KGB) dataset [24] contains five gait sequences for each of the 164 participants, who walked from left to right in a clockwise direction and returned in the opposite direction. An X-Box 360 Kinect sensor was used to collect the walking sequences of both males and females ranging from 17 to 45 years old. College students were the major contributing volunteers in the KGB dataset.

The gait cycle detection algorithm is applied to detect multiple gait cycles from the gait sequences of both datasets. The 3D matrix generation method is applied to prepare the input of 3D matrices from the gait cycles. The proposed residual learning-based KinectGaitNet model is trained using the samples of 3D matrices, and optimized weights are stored after the registration process. The optimized weights of the trained KinectGaitNet model are used in the identification phase. Five-fold cross-validation is conducted on both datasets, since five gait sequences of each of the participants of both datasets are available. Therefore, the proposed model can be evaluated by every gait sequences of each of the individuals to show the fairness of the model for each of the sets.

### 4.1. Performance of Optimization Method and Batch Size

The weights of the proposed CNN architecture are optimized using the Adam optimization method. Furthermore, Root Mean Square Propagation (RMSProp) and Stochastic Gradient Descent (SGD) optimization methods are applied to optimize the categorical cross-entropy objective function. The performances of the three optimization methods are compared in terms of recognition accuracy, precision, recall, and F-score. Table 1 shows the average recognition results of the SGD, RMSProp, and Adam optimization methods on the UPCV dataset. The proposed 3D matrix generation and KinectGaitNet with ReLU activation and SGD optimizer achieve the lowest recognition accuracy of 80.63%. RMSProp improves accuracy by 15% over SGD. The recognition accuracy, precision, recall, and F-score of 96.91%, 96.66%, 96.17%, and 96.02%, respectively, are achieved using the Adam optimizer. Thus, the Adam optimizer provides the best recognition performance on the UPCV dataset.

The average recognition performance of the proposed CNN model with the ReLU activation function and different optimization methods on the KGB dataset is shown in Table 2. The performances of the SGD, RMSProp, and Adam optimizers are close to each other. The recognition accuracies of 99.25%, 99.27%, and 99.33% are achieved by the SGD, RMSProp, and Adam optimization methods, respectively. The Adam optimizer again secures the best recognition accuracy, precision, recall, and F-score on the KGB dataset.

It is worth pointing out that the batch size of 32 is used in the experiments mentioned in Table 1 and Table 2. Therefore, it is studied further to finalize the contribution of the batch size hyperparameter while training the proposed CNN architecture. Since the Adam optimizer minimizes the objective function better than SGD and RMSProp, an experiment on the batch size is performed using the Adam optimizer and ReLU activation function. Table 3 shows the average recognition performance of the proposed CNN model on the UPCV dataset trained using different batch sizes. Similar experiments using different batch sizes are also conducted on the KGB dataset (see Table 4). It is evident from Table 3 and Table 4 that the recognition performance is gradually decreased if the batch size is increased from 32 to 128. The accuracies of 3.11% and 0.14% are decreased on the UPCV and KGB datasets, respectively. Therefore, the performance of the proposed CNN model is best when the proposed CNN model is optimized by the Adam optimizer and is trained using a batch size of 32.

### 4.2. Performance of Pooling Method

The proposed method avoids resampling of the gait cycles to a fixed length. The Global Average Pooling (GAP) method is proposed to be added after the fifth residual block to handle a variable dimensional feature map and make the variable representation of the feature map to a fixed-length representation. Activations of different feature maps are accumulated using the GAP layer. The performance of the GAP is compared with Spatial Pyramid Pooling (SPP) [38] and Global Max Pooling (GMP) [39] methods. While using an SPP layer in the architecture, we add three levels of pyramid-wise pooling regions of 1, 2, and 4 size. The performance of the SPP, GMP, and GAP on the UPCV dataset is shown in Table 5. The performance of the SPP layer is the lowest among the three pooling methods. GMP achieves around a 5% better identification rate than SPP. However, GAP secures the highest recognition accuracy of 96.91%, precision of 96.66%, recall of 96.17%, and F-score of 96.02%.

On the KGB dataset, KinectGaitNet achieves 94.95% and 98.85% recognition accuracies with the SPP and GMP layers, respectively. The proposed architecture with the GAP layer has the higher accuracy, precision, recall, and F-score among these three pooling methods on the KGB dataset (see Table 6). Therefore, it is evident that KinectGaitNet with a GAP layer provides the best performance on both datasets. Furthermore, KinectGaitNet has the characteristics of handling variable dimensional gait cycles of individuals without the necessity of resampling the gait cycle to a fixed length.

### 4.3. Analysis of Training and Validation

**Figure 5:**
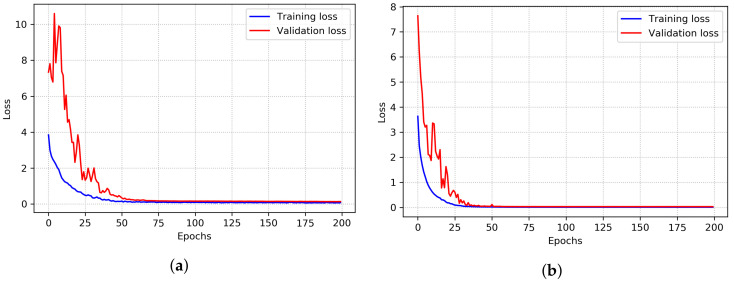
The average training accuracy and validation accuracy of KinectGaitNet on the UPCV and KGB datasets. (**a**) On UPCV dataset. (**b**) On KGB dataset.

The training loss and validation loss over epochs demonstrate whether the model is generalizing or memorizing. If the training loss and validation loss gradually decrease over epochs, a generalized pattern is learned by the model. Thus, the model overfitting can be identified from the learning curve. Since a five-fold cross-validation experiment is conducted, the average training loss and validation loss of the KinectGaitNet on the UPCV dataset are shown in Figure 5a. As the training loss and validation loss decrease, the training accuracy and validation accuracy increase gradually.

Figure 5b shows the average training loss and validation loss of the proposed CNN model on the KGB dataset. From Figure 5a,b, it can be pointed out that the trend of validation loss is downward over the epochs. Moreover, validation loss does not increase after it reaches a plateau, and there is no overfitting. Similar to validation loss, training loss does not show an upward trend after reaching a plateau on both datasets. Thus, the degradation problem is absent in the proposed deep learning architecture. Additionally, the experimental result supports that KinectGaitNet shows fairness to each of the training and test sets and does not suffer from overfitting.

### 4.4. Overall Performance Comparison

**Figure 6:**
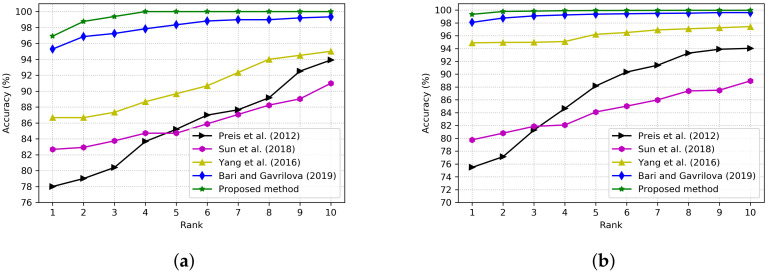
Performance comparison of the CMC scores of the proposed method with the methods [4,5,23,26] on the UPCV and KGB datasets. (**a**) On UPCV dataset. (**b**) On KGB dataset.

The performance of the proposed CNN model is evaluated using the Cumulative Match Characteristic (CMC) curve. The CMC curve of the proposed method on the UPCV dataset is shown in Figure 6a. Rank-1 recognition accuracy starts with 96.91% and reaches 100% at rank-4 on the UPCV dataset. On the other hand, the rank-1 recognition accuracy of the proposed method on the KGB dataset is 99.33%. It approaches 99.97% at rank-10 (see Figure 6b).

The performance of the proposed method is compared with the methods [4,5,23,26]. Comparisons of rank-1 to rank-10 CMC scores of the proposed method with the studied prior research on the UPCV dataset and KGB dataset are shown in Figure 6a,b, respectively. The proposed method achieves 100% accuracy at rank-4 on the UPCV dataset, whereas prior research can not achieve 100% accuracy at rank-10. On the other hand, on the KGB dataset, rank-1 recognition accuracy is 99.33%. The recognition accuracy of 99.98% is achieved at rank-10, whereas 88.95%, 94.03%, 97.42%, and 99.60% recognition accuracies are achieved at rank-10 by the state-of-the-art methods [4,5,23,26], respectively. In summary, the proposed method achieves a higher identification rate at each rank than the comparators on both datasets.

**Figure 7:**
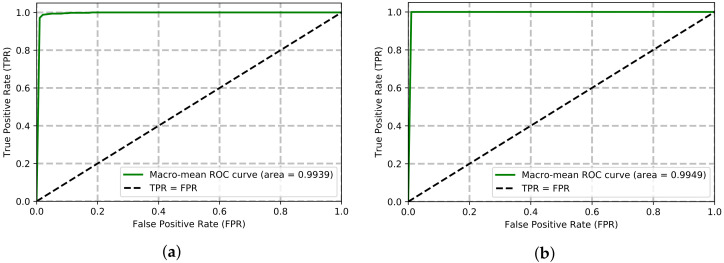
ROC curves on the UPCV and KGB datasets. (**a**) On UPCV dataset. (**b**) On KGB dataset.

The Receiver Operating Characteristic (ROC) is a performance metric for the classification. The probability measurement in the True Positive Rate (TPR) vs. False Positive Rate (FPR) shows the separability among the classes. Since five-fold cross-validation is conducted in our experiment, the macro-average is calculated to plot the ROC curve. The ROC curve on the UPCV dataset is showin in Figure 7a. The normalized area under the curve (nAUC) of ROC is 0.9936 on the UPCV dataset. On the other hand, the ROC curve on the KGB dataset is shown in Figure 7b. The nAUC score of the proposed method is 0.9949 on the KGB dataset.

On the other hand, Equal Error Rate (EER) is another performance metric for the biometric security. The smaller the EER score is, the better the verification system. The EER scores of the proposed method on UPCV and KGB datasets are 0.0202 and 0.0101, respectively. The nAUC and EER scores of the proposed Kinect-based gait recognition method on both datasets are compared with four prior works (see Table 7 and Table 8). The proposed method secures the highest nAUC score and the lowest EER score against all the prior research studied in this research on both datasets.

Trainable and non-trainable parameters are determined to calculate the total parameter count of the proposed CNN architecture. Since there are 30 participants in the UPCV dataset and 164 participants in the KGB dataset, a fully connected layer of 30 nodes and 164 nodes is added after the global average pooling layer for the identification in the UPCV and KGB datasets, respectively. The total count of the model parameters on both datasets is shown in Table 9. The total number of parameters of the method [23] is 4496926 on the UPCV dataset and 4514212 on the KGB dataset. Therefore, the proposed KinectGaitNet has over nine times fewer parameters than the architecture in method [23].

The system configurations for determining the inference time of the proposed CNN model are Intel Core i7-8700 CPU of 3.20 GHz, 16 GB of RAM, and GPU of NVIDIA GeForce GTX 1080. The running time of identification of a 3D matrix to a person is $3.30\times 10^{-4}$ s, whereas the running time of prediction by the method [23] is $3.85\times 10^{-4}$ s. Therefore, the proposed method is 14.3% faster than method [23].

### 4.5. Comparison with State-of-the-Art Works

Prior works, such as those of Ball et al. [33], Preis et al. [4], Ahmed et al. [25], Sun et al. [5], and Yang et al. [26], proposed handcrafted features to train the machine learning model for Kinect-based gait recognition. More recently, Bari and Gavrilova [23] introduced two unique geometric features to train their proposed deep learning neural network (DLNN) architecture. Our proposed method does not introduce handcrafted features; rather, it introduces the 3D matrix generation method to prepare the coordinates of the body joints for the training of the residual learning-based convolutional neural network. The proposed CNN model extracts optimized hierarchical features using the backpropagation algorithm. Performance comparison of the proposed method with prior research on the UPCV and KGB datasets is shown in Table 10 and Table 11, respectively.

Joint relative cosine dissimilarity (JRCD) and joint relative triangle area (JRTA) [23] provide better recognition results than prior research. The KinectGaitNet achieves higher recognition results than [23] in terms of accuracy, precision, recall, and F-score. On both the UPCV and KGB datasets, the proposed CNN architecture with ReLU activation, global average pooling, and Adam optimizer secures higher accuracy than [23] with nine times fewer parameters and 14.3% faster inference time. It also extracts better distinctive features than the traditional machine learning models introduced in [4,5,23,26,33]. Table 10 and Table 11 support the aforementioned claim. The KinectGaitNet secures the best recognition performance on both benchmark datasets. On the UPCV dataset, the highest recognition accuracy, precision, recall, and F-score are 96.91%, 96.66%, 96.17%, and 96.02%, respectively. On the KGB dataset, the highest recognition accuracy, precision, recall, and F-score are 99.47%, 99.49%, 99.49%, and 99.48%, respectively. Moreover, the CMC scores are better at each rank, the normalized area under the curve is higher, and the equal error rate is lower than in prior research.

## 5. Conclusions and Future Work

In this paper, the residual learning-based convolutional neural network KinectGaitNet is proposed for Kinect-based gait recognition. A new 3D matrix generation algorithm is proposed. Resampling of the gait cycle to a fixed length is avoided using the global average pooling layer in the KinectGaitNet. The proposed method is evaluated on two benchmark datasets of Kinect-based gait recognition. On the UPCV and KGB datasets, 96.91% and 99.33% accuracies are achieved, respectively. The achieved recognition performance is superior to all recently proposed state-of-the-arts methods. The running time of prediction is 14.3% faster, and the parameter count is reduced by 89.03% over the most recent method. This performance is achieved because of the proposed residual learning-based CNN architecture, no resampling of the gait cycle, and absence of the handcrafted features. In the future, the effect of the missing body joints will be investigated to develop the gait recognition system applicable to real-life scenarios. An improved CNN architecture for better hierarchical feature extraction can be the extension of the proposed method. Finally, experimentation with different walking trajectories and testing the performance of the method under varied clothing conditions can be another future research direction.

## Figures and Tables

**Table 1:** Average recognition performance of the proposed convolutional neural network with ReLU activation function and different optimization methods on the UPCV dataset. The best performance is shown in bold.

Optimizers	Accuracy	Precision	Recall	F-Score
SGD	80.63	73.88	74.87	72.69
RMSProp	95.41	94.87	94.43	94.01
**Adam**	**96.91**	**96.66**	**96.17**	**96.02**

**Table 2:** Average recognition performance of the proposed convolutional neural network with the ReLU activation function and different optimization methods on the KGB dataset. The best performance is shown in bold.

Optimizers	Accuracy	Precision	Recall	F-Score
SGD	99.25	99.27	99.25	99.25
RMSProp	99.27	99.31	99.31	99.27
**Adam**	**99.33**	**99.36**	**99.35**	**99.33**

**Table 3:** Average recognition performance of the proposed KinectGaitNet with ReLU activation functions, Adam optimizer, and different batch sizes on the UPCV dataset. The best performance is shown in bold.

Batch Size	Accuracy	Precision	Recall	F-Score
**32**	**96.91**	**96.66**	**96.17**	**96.02**
64	94.75	93.02	92.06	92.26
128	93.80	92.88	91.67	91.74

**Table 4:** Average recognition performance of the proposed KinectGaitNet with ReLU activation functions, Adam optimizer, and different batch sizes on the KGB dataset. The best performance is shown in bold.

Batch Size	Accuracy	Precision	Recall	F-Score
**32**	**99.33**	**99.36**	**99.35**	**99.33**
64	99.30	99.34	99.35	99.31
128	99.19	98.96	98.92	98.93

**Table 5:** Average recognition performance of KinectGaitNet with different pooling methods on the UPCV dataset. The best performance is shown in bold.

Pooling	Accuracy	Precision	Recall	F-Score
Spatial Pyramid	80.37	80.73	77.76	76.74
Global Max	85.58	83.48	83.15	81.56
**Global Average**	**96.91**	**96.66**	**96.17**	**96.02**

**Table 6:** Average recognition performance of KinectGaitNet with different pooling methods on the KGB dataset. The best performance is shown in bold.

Pooling	Accuracy	Precision	Recall	F-Score
Spatial Pyramid	94.95	95.68	95.64	95.07
Global Max	98.85	98.92	98.93	98.87
**Global Average**	**99.33**	**99.36**	**99.35**	**99.33**

**Table 7:** nAUC and EER of the proposed method and the prior works on the UPCV dataset. The best performance is shown in bold.

Method	nAUC	EER
Sun et al. [5]	0.7866	0.2929
Yang et al. [26]	0.9285	0.1212
Preis et al. [4]	0.9475	0.1010
Bari and Gavrilova [23]	0.9927	0.0202
**Proposed method**	**0.9939**	**0.0202**

**Table 8:** nAUC and EER of the proposed method and the prior works on the KGB dataset. The best performance is shown in bold.

Method	nAUC	EER
Sun et al. [5]	0.9038	0.1717
Preis et al. [4]	0.9279	0.1010
Yang et al. [26]	0.9696	0.0505
Bari and Gavrilova [23]	0.9946	0.0101
**Proposed method**	**0.9949**	**0.0101**

**Table 9:** Parameter count of the KinectGaitNet.

Type of the Parameters	UPCV Dataset	KGB Dataset
Trainable parameter	469438	478148
Non-trainable parameter	2464	2464
Total	471902	480612

**Table 10:** Performance comparison of the proposed method with prior research on the UPCV dataset. The best performance is shown in bold.

Methods	Accuracy	Precision	Recall	F-Score
Ball et al. [33]	57.00	53.19	57.87	51.32
Preis et al. [4]	78.00	74.27	73.41	70.43
Sun et al. [5]	82.67	80.50	80.19	79.67
Yang et al. [26]	86.67	85.48	83.76	83.08
[25] + (DLNN + tanh + Adam [23])	93.33	91.15	90.70	89.73
Bari and Gavrilova [23]	95.30	94.40	94.02	93.27
**KinectGaitNet + ReLU + Adam**	**96.91**	**96.66**	**96.17**	**96.02**

**Table 11:** Performance comparison of the proposed method with prior research on the KGB dataset. The best performance is shown in bold.

Methods	Accuracy	Precision	Recall	F-Score
Ball et al. [33]	37.55	37.84	38.11	34.25
Preis et al. [4]	75.46	77.70	75.34	73.71
Sun et al. [5]	79.76	80.12	79.32	75.74
Yang et al. [26]	94.88	94.67	95.02	93.92
[25] + (DLNN + tanh + Adam [23])	95.62	95.92	95.94	95.14
Bari and Gavrilova [23]	98.08	98.00	98.26	97.81
**KinectGaitNet + ReLU + Adam**	**99.33**	**99.36**	**99.35**	**99.33**

## Data Availability

Not applicable.
